# Supplementary material for: Isolation and characterisation of novel phages infecting Lactobacillus plantarum and proposal of a new genus, “Silenusvirus”
Source: Sci Rep. 2020 May 29;10:8763. doi: 10.1038/s41598-020-65366-6 (PMC7260188; doi:10.1038/s41598-020-65366-6)
Supplement: Supplementary file 1 — Supplementary Information. [file 41598_2020_65366_MOESM1_ESM.pdf]

Isolation and characterisation of novel phages infecting *Lactobacillus plantarum* and proposal of a new genus, "Silenusvirus"

Ifigeneia Kyrkou[1,2], Alexander Byth Carstens[1,3], Lea Ellegaard-Jensen[1], Witold Kot[1,3], Athanasios Zervas[1], Amaru Miranda Djurhuus[1,3], Horst Neve[4], Charles M.A.P. Franz[4], Martin Hansen[1], and Lars Hestbjerg Hansen[1,3,*]

[1]Department of Environmental Science, Aarhus University, Frederiksborgvej 399C, Roskilde, 4000, Denmark
[2]Department of Clinical Microbiology, Rigshospitalet, Copenhagen, 2100, Denmark
[3]Department of Plant and Environmental Sciences, University of Copenhagen, Thorvaldsensvej 40, Frederiksberg, 1871, Denmark
[4]Department of Microbiology and Biotechnology, Max Rubner-Institut, Hermann-Weigmann-Straße 1, Kiel, 24103, Germany
[*]lhha@plen.ku.dk

Supplementary information

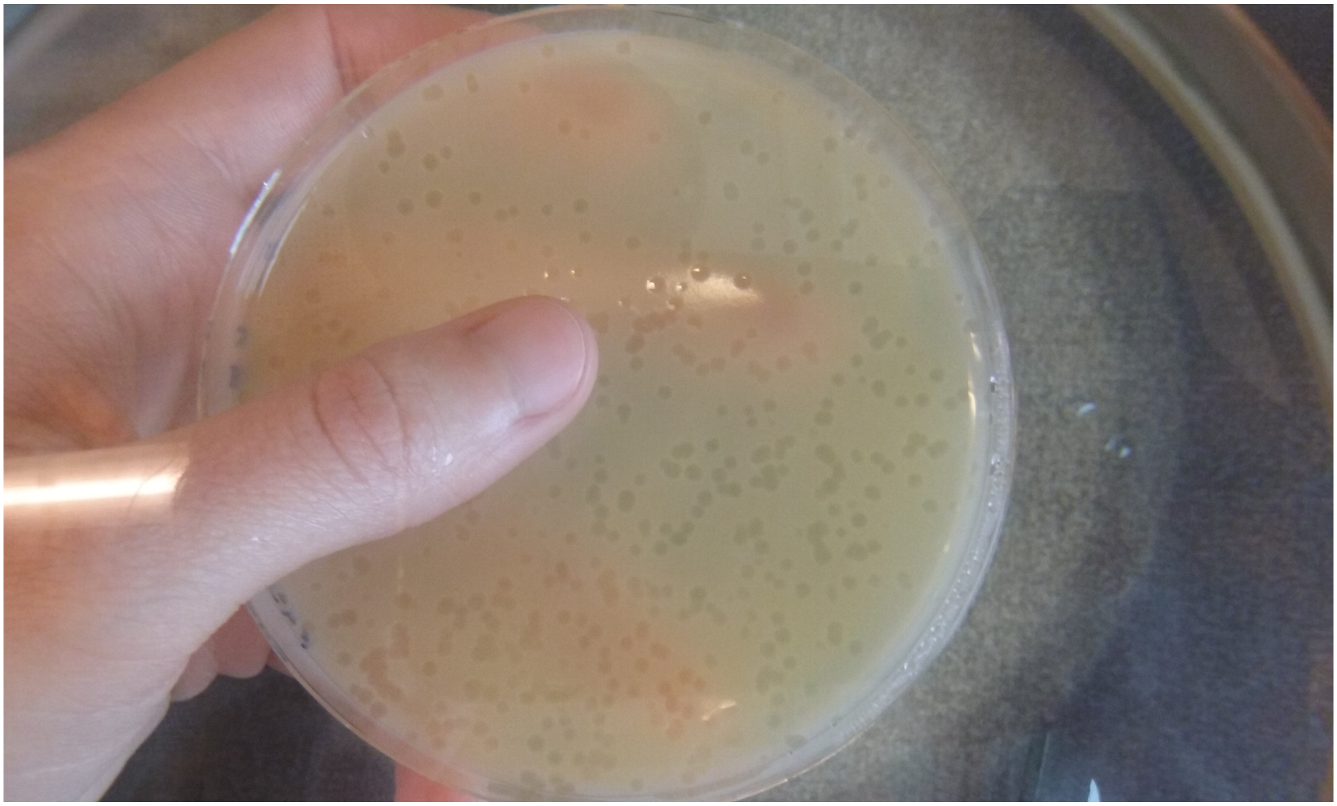

Figure 1. Plaques produced on a lawn of *L. plantarum* L1 by phage Bassarid.

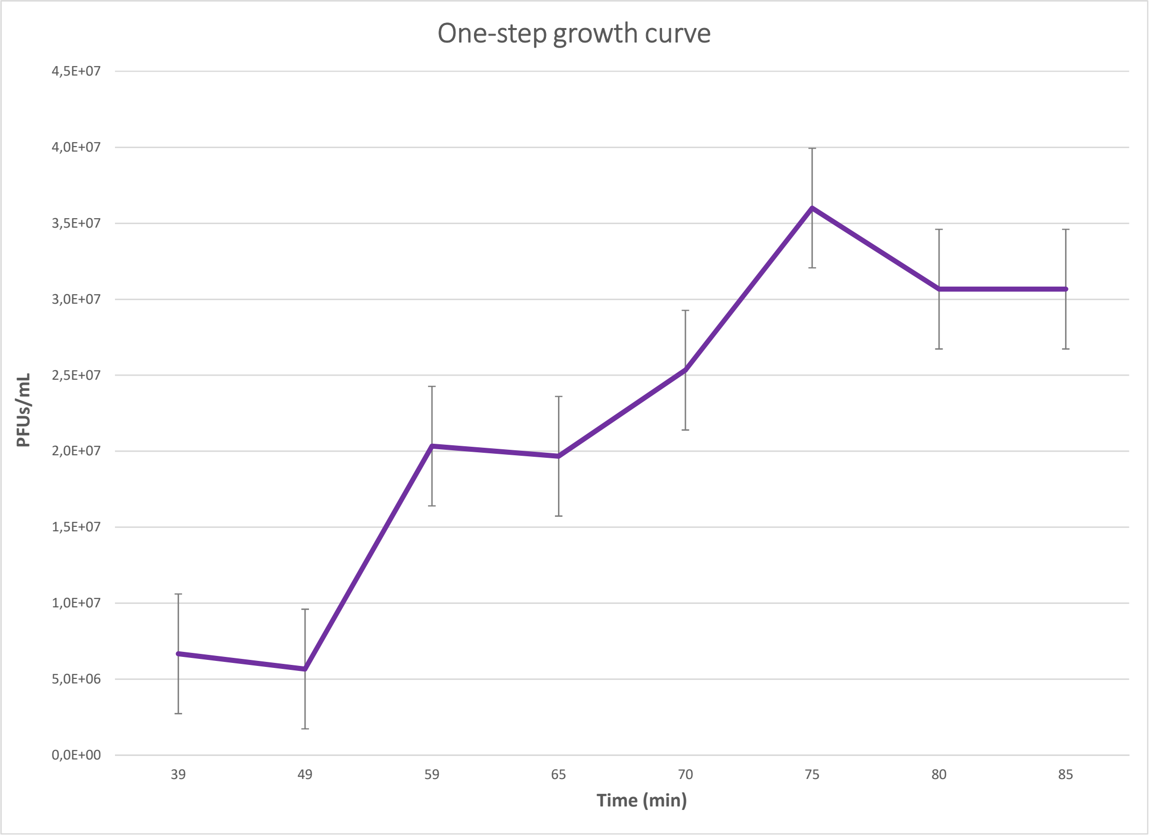

Figure 2. One-step growth curve of phage Silenus.

Time (min)	Average Adsorbed (PFUs/mL)	Adsorbed Triplicate A (PFUs/mL)	Adsorbed Triplicate B (PFUs/mL)	Adsorbed Triplicate C (PFUs/mL)	Unadsorbed Triplicate A (PFUs/mL)	Unadsorbed Triplicate B (PFUs/mL)	Unadsorbed Triplicate C (PFUs/mL)
39	6.7×10^6	4.0×10^6	9.0×10^6	7.0×10^6	10^4	6.0×10^3	1.3×10^4
49	5.7×10^6	4.0×10^6	5.0×10^6	8.0×10^6	-	-	-
59	2.0×10^7	2.6×10^7	1.4×10^7	2.1×10^7	-	-	-
65	2.0×10^7	2.0×10^7	1.5×10^7	2.4×10^7	-	-	-
70	2.5×10^7	2.0×10^7	1.8×10^7	3.8×10^7	-	-	-
75	3.6×10^7	3.7×10^7	3.0×10^7	4.1×10^7	-	-	-
80	3.1×10^7	2.7×10^7	3.7×10^7	2.8×10^7	-	-	-
85	3.1×10^7	3.0×10^7	3.6×10^7	2.6×10^7	-	-	-

Table 1. Raw data on phage adsorption and growth kinetics for phage Silenus.

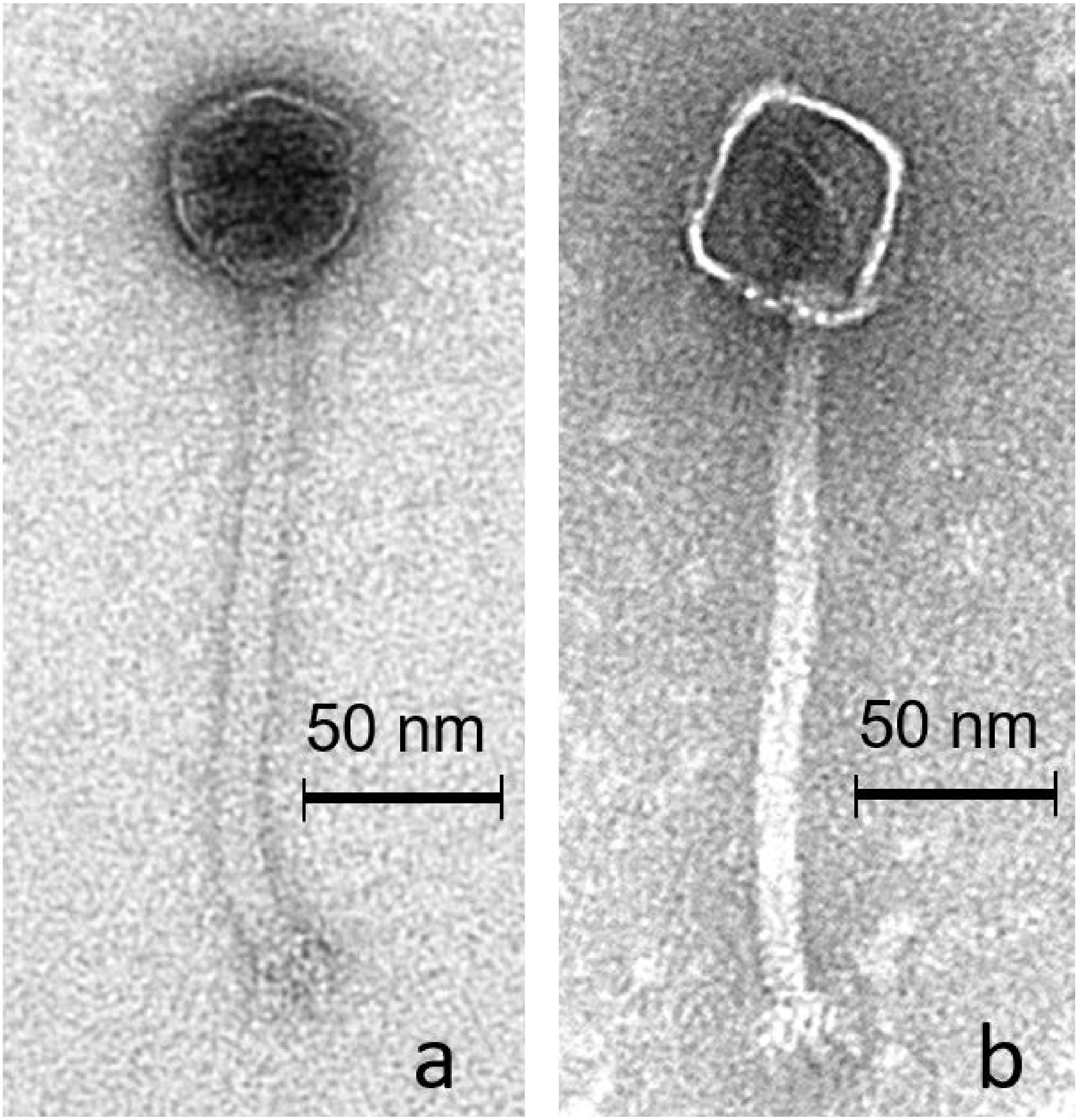

Figure 3. TEM micrographs of phages Sabazios (a) and Bassarid (b). For phage Bassarid, only phage particles with defective (empty) capsids could be detected. The dimensions of these phages' virions are shown in Table S2.

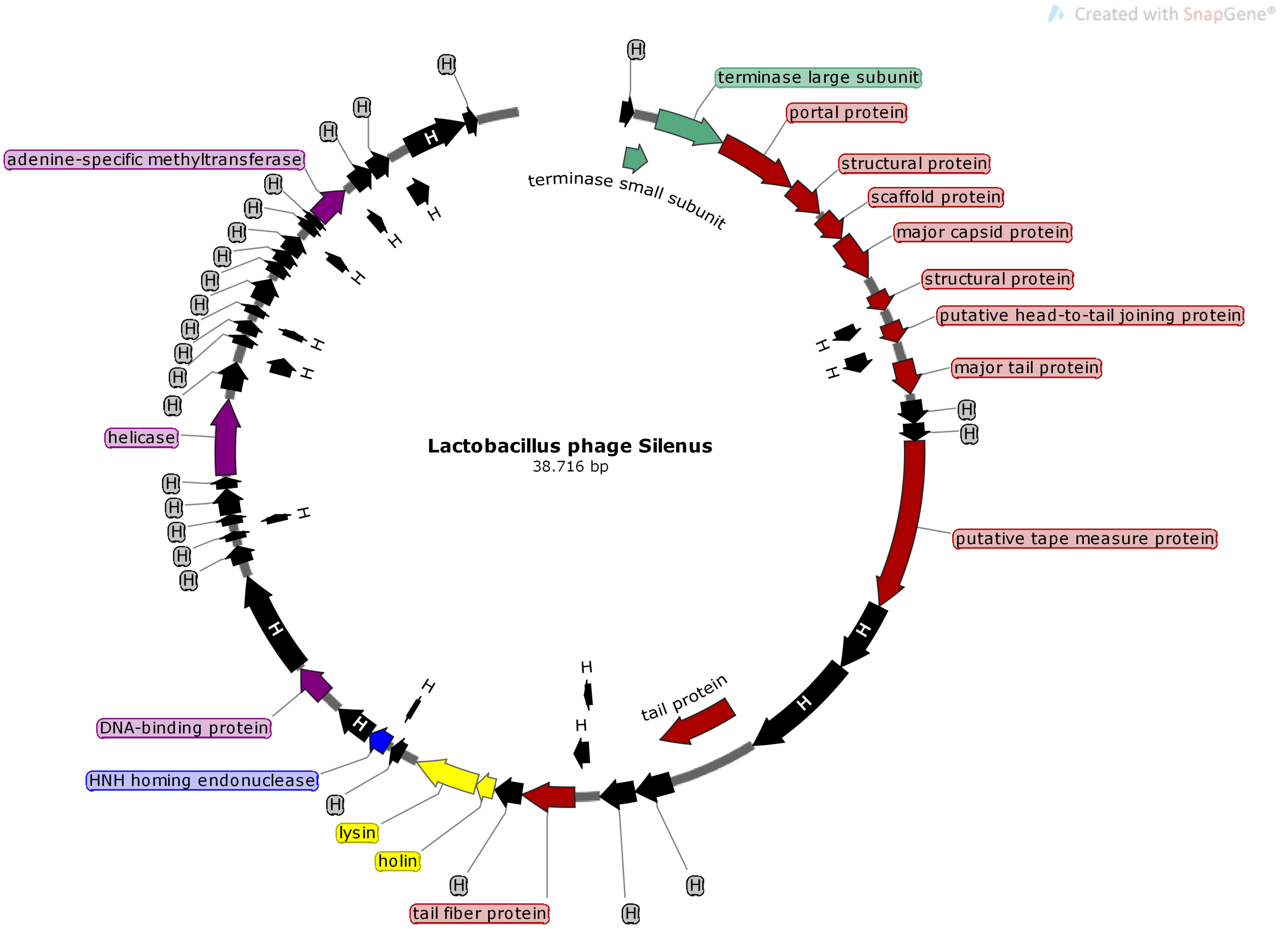

Figure 4. Genome map featuring all predicted proteins with assigned functions for phage Silenus. Colour code corresponds to the same protein functions as in Fig. 5.

Phage Sabazios	Dimensions (nm)	Counted Phage Particles
**Head Diameter**	54.5 ± 2.4	18
**Tail Length (with Baseplate)**	173.1 ± 6.1	18
**Tail Width**	11.5 ± 0.5	18
**Baseplate Width**	17.6 ± 1.3	18
**Baseplate Length**	11.3 ± 1.1	18
**Collar Width**	14.9 ± 0.4	3

Phage Bassarid	Dimensions (nm)	Counted Phage Particles
**(Empty) Head Diameter**	59.2 ± 1.2	4
**Tail Length (with Baseplate)**	177.0 ± 1.4	4
**Tail Width**	11.7 ± 0.5	4
**Baseplate Width**	18.7 ± 2.2	4
**Baseplate Length**	could not be measured	could not be measured

Table 2. TEM analysis results of virion dimensions for phages Sabazios and Bassarid. For the TEM micrographs of these phages see Fig. S3.

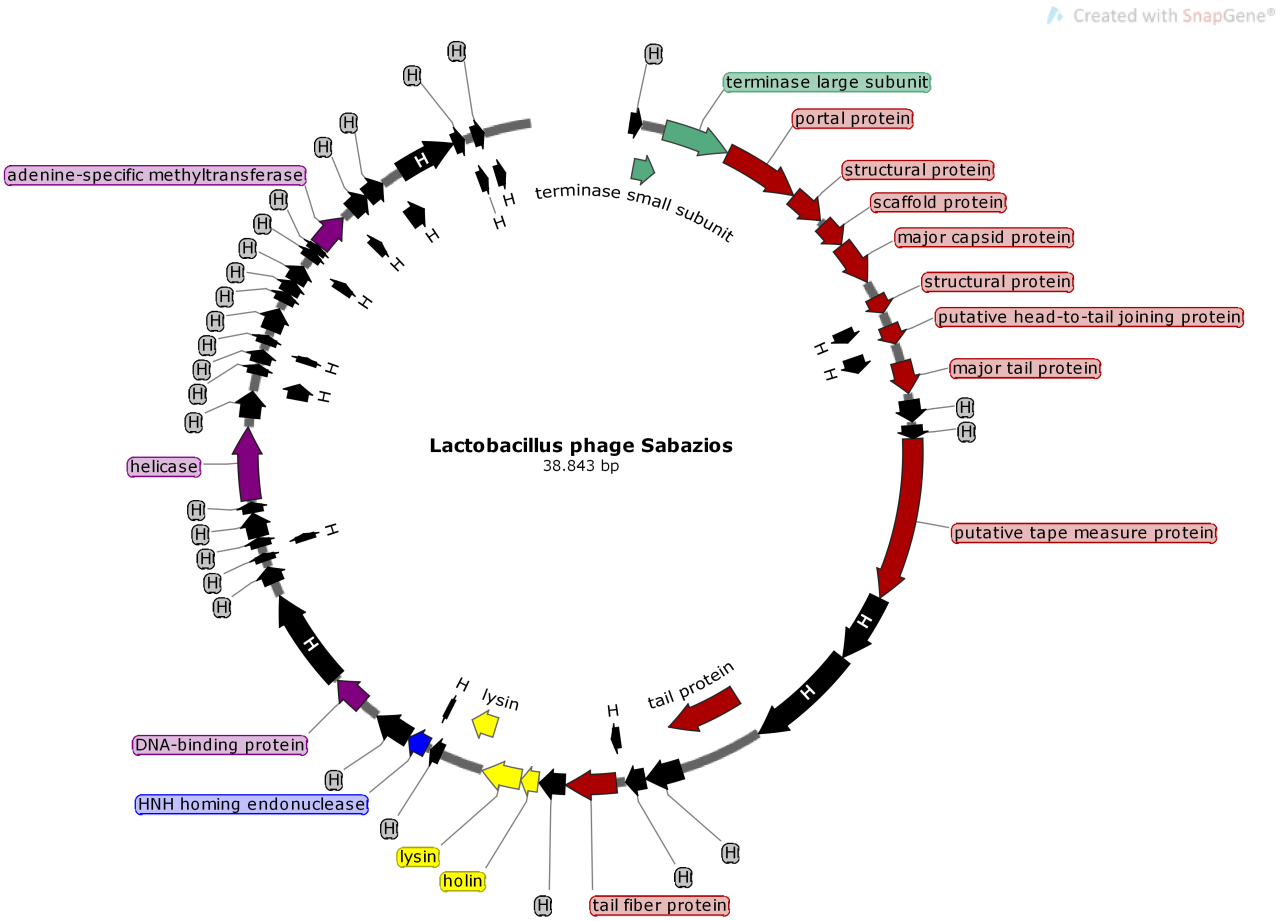

Figure 5. Genome map featuring all predicted proteins with assigned functions for phage Sabazios. Colour code corresponds to the same protein functions as in Fig. 5.

Description	Function	Coverage (%)	No of Peptides	Peptide Spectrum Matches	Molecular Mass (kDa)	Gene
Major capsid protein	Structural	12	3	40	31.2	peg. 14
Putative head-to-tail joining protein	Structural	1	1	30	62.5	peg. 11
Hypothetical protein	Structural	3	1	15	49.7	peg. 5
Adenine-specific methyltransferase	DNA modification	6	1	25	14.2	peg. 27
Hypothetical protein	Unknown	2	1	1	73.1	peg. 48

Table 3. Results of the protein sequencing analysis for phage Silenus.

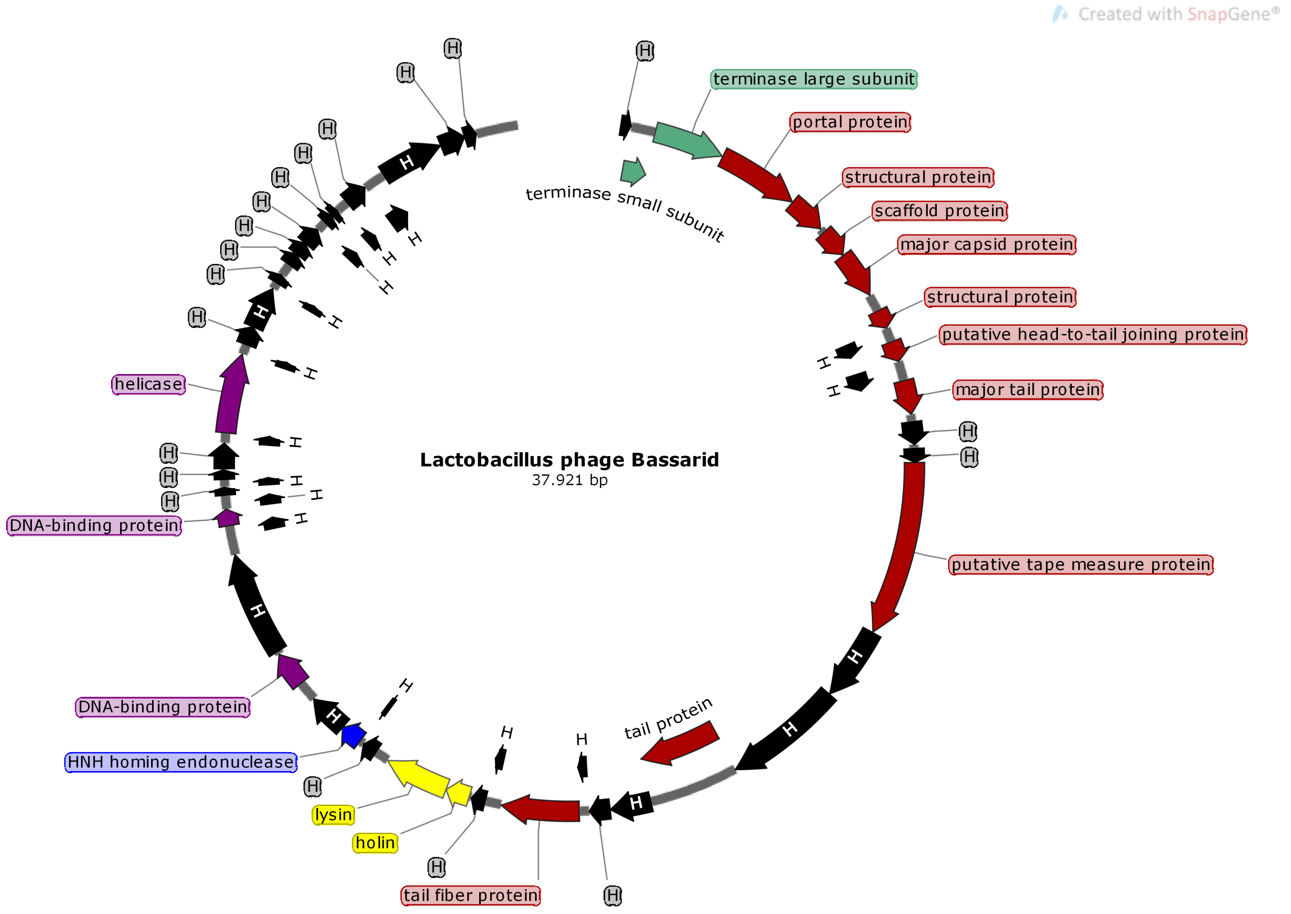

Figure 6. Genome map featuring all predicted proteins with assigned functions for phage Bassarid. Colour code corresponds to the same protein functions as in Fig. 5.

Bacteriophage record	NCBI nucleotide accession number
Lactobacillus phage c5	NC_019449.1
Lactobacillus phage Ld3	NC_025421.1
Lactobacillus phage Ld17	NC_025420.1
Lactobacillus phage Ld25A	NC_025415.1
Lactobacillus phage LL-Ku	NC_022989.1
Lactobacillus phage phiLdb	NC_022762.1
Lactobacillus phage phiJL-1	NC_006936.1
Lactobacillus phage ATCC 8014-B1	NC_019916.1
Pediococcus phage clP1	NC_016161.1
Bacillus phage SPP1	NC_004166.2
Lactobacillus phage A2	NC_004112.1
Lactobacillus phage J-1	NC_022756.1
Lactobacillus phage P1174	MG913376.1
Lactobacillus phage CL1	NC_028888.1
Lactobacillus phage CL2	NC_028835.1
Lactobacillus phage iLp1308	NC_028911.1
Oenococcus phage phiOE33PA	MH220877.1

Table 4. NCBI nucleotide accession numbers of all available phage records cited by this article.

```
Reference sequence (1): AUV59728.1
Identities normalised by aligned length.
Colored by: identity

                       cov    pid   1 [        .         .         .         .         :         .         .         . 80
1 AUV59728.1       100.0% 100.0%     MKNKLLLLAASLSLFLMPLTAMASKGDQGVDWAKYQGTNGVFGYSSDKFVISQLGGTVNGSIYEQYTYPTQVASAIAAGK
2 AYH91857.1        64.8%  92.2%     MKKKLLLLLASLALFLMPLTAMASKGDQGVDWAKYQGTNGVFGYSKDKFVISQLGGTVNGSIYEQYTYPTQVASAIAAEK
3 AVH85737.1       100.0%  93.5%     MKKKLLLLLASLALFLMPLTAMASKGDQGVDWAKYQGTNGVFGYSKDKFVISQLGGTVNGSIYEQYTYPTQVASAIAAEK
4 AYH91858.1        46.5%  93.0%     --------------------------------------------------------------------------------
  consensus/100%                     ................................................................................
  consensus/90%                      ................................................................................
  consensus/80%                      ................................................................................
  consensus/70%                      MKpKLLLLhASLuLFLMPLTAMASKGDQGVDWAKYQGTNGVFGYSpDKFVISQLGGTVNGSIYEQYTYPTQVASAIAAtK

                       cov    pid  81          .         1         .         .         .         .         :         . 160
1 AUV59728.1       100.0% 100.0%     RAHTYLWGQFGSSKTQAKAMLDYMLPKVQTPKGSIVALDYEDGASGDKQANTDTIKYALKRIKDFGYTPMLYGYLKYFNA
2 AYH91857.1        64.8%  92.2%     RAHTYLWGQFGSSKAQAKAMLDYMLPKVQTPKGSIVALDYEDGASGDKQANTDAILYALQRIKNAGYTPMLYGYLNYFNA
3 AVH85737.1       100.0%  93.5%     RAHTYLWGQFGSSKAQAKAMLDYMLPKVQTPKGSIVALDYEDGASGDKQANTDAILYALQRIKNAGYTPMLYGYLNYFNA
4 AYH91858.1        46.5%  93.0%     --------------------------------------------------------------------------------
  consensus/100%                     ................................................................................
  consensus/90%                      ................................................................................
  consensus/80%                      ................................................................................
  consensus/70%                      RAHTYLWGQFGSSKsQAKAMLDYMLPKVQTPKGSIVALDYEDGASGDKQANTDsIhYALpRIKshGYTPMLYGYLpYFNA

                       cov    pid 161          .         .         .         2         .         .         .         . 240
1 AUV59728.1       100.0% 100.0%     HVYLSQISGTYKLWLGEYPDYNVTPKPNYNYFPSWENVALFQFTSTYIAGGLDGNIDLTGITDNGYTKNNKPVTNTPAVD
2 AYH91857.1        64.8%  92.2%     HVYLGQISKTYKLWLGEYPDYNVTPKPNYNYFPSWENVALFQFTSTYIAGGLDGNIDLTGITDNGYTKNNKPVTDTPAVD
3 AVH85737.1       100.0%  93.5%     HVYLGQISKTYKLWLGEYPDYNVTPKPNYNYFPSWENVALFQFTSTYIAGGLDGNIDLTGITDNGYTKNNKPVTDTPAVD
4 AYH91858.1        46.5%  93.0%     -----------------------------------------------------MNIDLTGITDNGYTKNNKPVTDTPAVD
  consensus/100%                     .....................................................hNIDLTGITDNGYTKNNKPVTsTPAVD
  consensus/90%                      .....................................................hNIDLTGITDNGYTKNNKPVTsTPAVD
  consensus/80%                      .....................................................hNIDLTGITDNGYTKNNKPVTsTPAVD
  consensus/70%                      HVYLuQIStTYKLWLGEYPDYNVTPKPNYNYFPSWENVALFQFTSTYIAGGLDGNIDLTGITDNGYTKNNKPVTDTPAVD

                       cov    pid 241          :         .         .         .         .         3         .         . 320
1 AUV59728.1       100.0% 100.0%     TGKEAEDTPKADIKKGDKVKVKFSAKHWATGEAIPSWVRGNTYTVTAVSGKKVLLSGINSWIARSNVEILQTNTVQSNVK
2 AYH91857.1        64.8%  92.2%     TGKDADETPKADIKKGEH--------------------------------------------------------------
3 AVH85737.1       100.0%  93.5%     TGKDADETPKADIKKGDKVKVKFSAKHWATGEAIPLWVRGNTYTVTAVSGKKVLLSGINSWIARSNVEILQNKPVQSNVK
4 AYH91858.1        46.5%  93.0%     TGKDADETPKADIKKGDKVKVKFSAKHWATGEAIPLWVRGNTYTVTAVSGKKVLLSGINSWIARSNVEILQNKPVQSNVK
  consensus/100%                     TGK-A--TPKADIKKG-+..............................................................
  consensus/90%                      TGK-A--TPKADIKKG-+..............................................................
  consensus/80%                      TGK-A--TPKADIKKG-+..............................................................
  consensus/70%                      TGKDADETPKADIKKGDKVKVKFSAKHWATGEAIP.WVRGNTYTVTAVSGKKVLLSGINSWIARSNVEILQspsVQSNVK

                       cov    pid 321          .         .         :         .         .         .         .       ] 398
1 AUV59728.1       100.0% 100.0%     LPSGVKRESGTFTANQTLRVWNKPGMSYTGVNYYRGESVRYQGYIRNGNYIYAAYQSSNGTWHYVAVRENGVALGTFK
2 AYH91857.1        64.8%  92.2%     ------------------------------------------------------------------------------
3 AVH85737.1       100.0%  93.5%     LPSGVKREYGTFTANTTLRVWNKPGMAYTGVNYYRGESVRYQGYIRNGNYIYAAYQSSNGAWHYVAVRENGVALGTFK
4 AYH91858.1        46.5%  93.0%     LPSGVKREYGTFTANTTLRVWNKPGMAYTGVNYYRGESVRYQGYIRNGNYIYAAYQSSNGAWHYVAVRENGVALGTFK
  consensus/100%                     ..............................................................................
  consensus/90%                      ..............................................................................
  consensus/80%                      ..............................................................................
  consensus/70%                      LPSGVKRE.GTFTANpTLRVWNKPGMuYTGVNYYRGESVRYQGYIRNGNYIYAAYQSSNGsWHYVAVRENGVALGTFK
```

MView 1.63, Copyright © 1997-2018 Nigel P. Brown

Figure 7. Multiple amino acid sequence alignments of the two Sabazios lysins (AYH91857.1 and AYH91858.1), the lysin of Bassarid (AUV59728.1) and the lysin of Silenus (AVH85737.1).

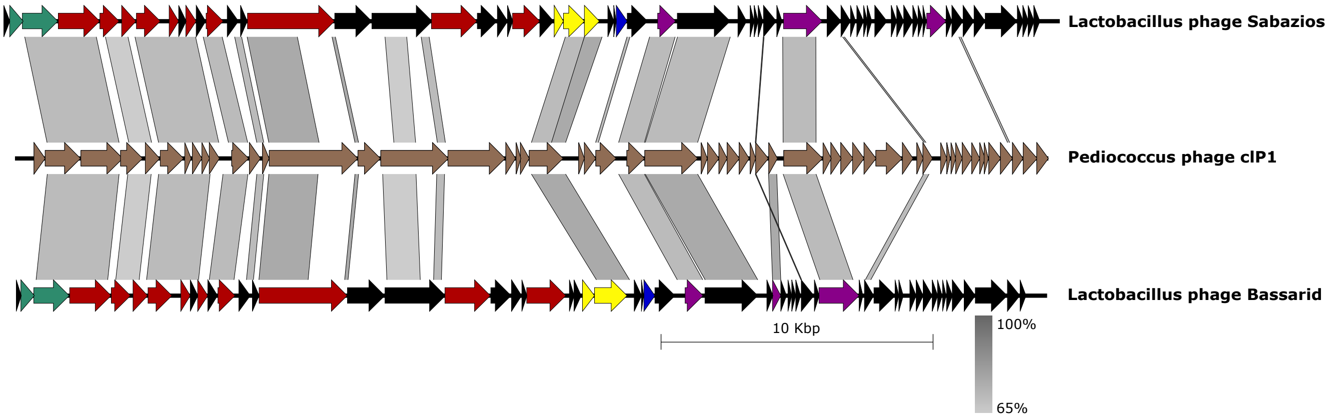

Figure 8. Genomic synteny comparisons with Easyfig and the BLASTn algorithm. The genomes of this study's phages Sabazios and Bassarid are compared to the distantly related Pediococcus phage clP1. Arrows represent the locations of coding sequences and shaded lines reflect the degree of homology between pairs of phages. Colours other than black mark specific predicted protein functions; DNA packaging is in turquoise, morphogenesis in red, lysis in yellow, selfish genetic elements in blue and metabolism/modification of nucleic acids in deep purple.
